# Wnt signaling inhibition confers induced synthetic lethality to PARP inhibitors

**DOI:** 10.15252/emmm.202114002

**Published:** 2021-03-30

**Authors:** Stephane Angers

**Affiliations:** ^1^ Leslie Dan Faculty of Pharmacy University of Toronto Toronto ON Canada; ^2^ Department of Biochemistry University of Toronto Toronto ON Canada

**Keywords:** Cancer, Regenerative Medicine

## Abstract

Despite considerable efforts, therapeutic strategies targeting the Wnt pathway are still not clinically available. The pervasive role of Wnt‐βcatenin signaling for the control of stem cells during normal tissue homeostasis makes the on‐target toxicity of available therapeutic grade molecules an important limitation preventing their clinical introduction. The article in this issue of *EMBO Molecular Medicine* by Kaur *et al* (2021) reveals that treatment of Wnt‐addicted cancer cells with inhibitors of Wnt signaling induces a state of BRCAness leading to hypersensitivity to PARP inhibitors. This is a new example of induced synthetic lethality that could pave the way for new indications for PARP inhibitors or may contribute to the long‐awaited clinical introduction of therapeutic agents targeting the Wnt pathway.

## Graphical abstract

S. Angers discusses a new example of induced‐synthetic lethality reported by Kaur *et al* (this issue of *EMBO Mol Med*) that shows that treatment of Wnt‐addicted cancer cells with inhibitors of Wnt signaling induces a state of BRCAness leading to hypersensitivity to PARP inhibitors.

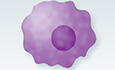

In recent years, the discovery that the E3 ubiquitin ligases RNF43/ZNRF3 function as R‐spondin co‐receptors and as functional negative regulators of the Wnt pathway (de Lau *et al,* 2014) provided a molecular basis underlying the occurrence of *RNF43* mutations and R‐spondin fusions in various cancers such as colorectal, pancreatic, ovarian, biliary tract, and gastric (Seshagiri *et al,* 2012; Giannakis *et al,* 2014). In these cancers, cells exhibit hyperactive Wnt‐βcatenin signaling and are addicted to the presence of circulating Wnt growth factors. Therapeutic opportunities for Wnt‐addicted cancers are the targeting of (i) Porcupine (PORCN) (Jiang *et al,* 2013), an acyltransferase enzyme responsible for the palmitoylation of Wnt proteins, and (ii) Frizzled receptors using inhibiting antibodies (Gurney *et al,* 2012; Steinhart *et al,* 2017). However, limiting dose toxicity is an inherent problem associated with widespread pathway inhibition.

The preferential therapeutic vulnerability of cells with a given genotype, for example, cancer cells, is derived from the genetic concept of synthetic lethality describing a context whereby mutation in either of two genes has little effect on cell viability, but mutations in both genes result in death (Huang *et al,* 2020). Perhaps most famously highlighting this paradigm is the exquisite sensitivity of cancer cells harboring mutations in the homologous recombination genes *BRCA1* and *BRCA2* to PARP inhibitors (Lord *et al,* 2015). The clinical efficacy of PARP inhibitors was rapidly demonstrated for tumor arising due to germ line mutations in *BRCA1* and *BRCA2* but also due to the presence of somatic mutations within several other genes involved in homologous recombination repair (HRR) pathways such as ATM, ATR, PALB2, and the Fanconi anemia genes that commonly arise in a wide variety of tumors. This state of impaired HRR activity, which predicts the efficacy of PARP inhibitors, is commonly referred to as “BRCAness” as it phenocopies to some extent the germ line inactivation of the *BRCA1/2* genes (Lord & Ashworth, 2016).

In this issue of *EMBO Molecular Medicine*, Kaur *et al* (2021) performed a chemical screen to identify genes that synergize with Porcupine inhibitors in Wnt‐addicted cancers. The combination of the PARP inhibitor olaparib with the Porcupine inhibitor ETC‐159 revealed robust synergy in inhibiting the growth of various Wnt‐addicted cancer cell lines harboring mutations in *RNF43* or R‐spondin fusion. This was also validated *in vivo*, as co‐treatment with both inhibitors resulted in synergistic tumor growth inhibition when compared to the individual treatment.

Mechanistically, Wnt signaling inhibition was found to reduce the expression of several genes involved in DNA damage repair and more specifically genes important for HRR and Fanconi anemia pathways. The authors showed that Wnt pathway inhibition leads to impaired HRR efficiency, thereby creating a cellular state of “BRCAness” that underlies the hypersensitivity to PARP inhibitor. Importantly, treatment of cells with Porcupine inhibitors for 48 h led to strong decrease in HRR and Fanconi anemia pathway genes without altering the percentage of cells in S phase, confirming that the observed effect could not merely be attributed to the role of Wnt signaling during cell cycle progression. In addition, the authors described a signaling axis downstream of βcatenin regulating the levels of MYBL2. Depletion of MYBL2 in Wnt‐addicted cancer cells led to inhibition of HRR and FA genes expression, and MYBL2 was enriched at promoters of several DNA repair genes in a Wnt signaling‐dependent manner. Interestingly, MYBL2 was not enriched at promoters of canonical βcatenin target genes, such as *AXIN2*, putatively revealing a novel transcriptional axis downstream of Wnt pathway activation. Whether the regulation of DNA repair genes was directly regulated by βcatenin, required LEF/TCF factors, or required physical interactions of these factors with MYBL2 was however not demonstrated and remains to be established.

This study provides an example of “induced synthetic lethality”, defined when a first chemical treatment (in this case an inhibitor of Wnt signaling) induces a vulnerable state in tumor cells that can be exploited by a second treatment (in this case a PARP inhibitor) (Fig 1). While Porcupine inhibitors provide an effective way to block Wnt signaling in the context of *RNF43* mutations and R‐spondin fusions, they are ineffective in the context of tumors harboring more distal activating mutations in the pathway such as in *APC*, βcatenin, and *AXIN1* found for example in the majority of colorectal adenocarcinoma. Extending their observations, the authors showed that a tankyrase inhibitor, which leads to Axin stabilization and to inhibition of βcatenin signaling, also decreased expression of HRR genes in CRC cells and synergized with a PARP inhibitor, hinting that this particular induced synthetic lethality interaction may be a general phenomenon. In fact, when examining the intestinal stem cell niche, expression of HRR genes was associated with the stem and transit‐amplifying cells that exhibit high Wnt‐βcatenin signaling activity. While control of DNA repair by the Wnt‐βcatenin pathway may represent a mechanism to protect tissue stem cells from various insults, this raises a potential limitation of leveraging combined Wnt and PARP inhibition as a cancer therapy, since this may be predicted to also affect the normal tissues. However, it was previously described that the intestinal stem cell stroma, which secrete Wnt proteins, is especially resistant to Porcupine inhibition due to their high expression of ABC transporters (Chee *et al,* 2018). It is therefore conceivable that combining synergistic doses of Porcupine and PARP inhibitors could result in heightened cytotoxicity in the tumor cells while sparing normal cells and thereby increasing the therapeutic window.

**Figure 1:**
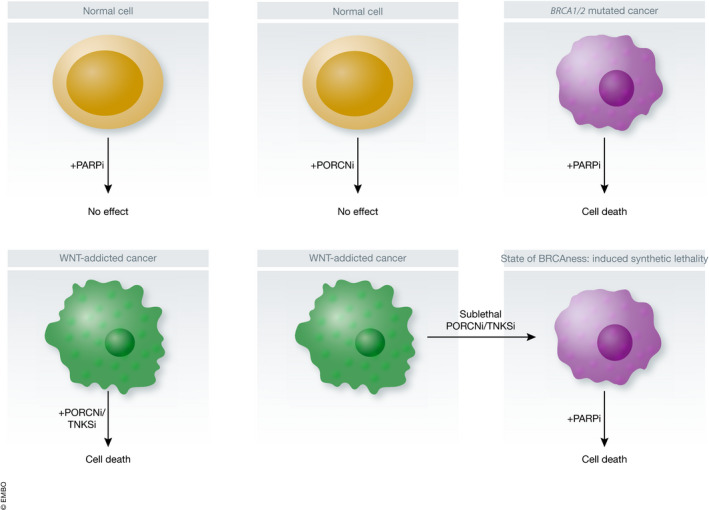
Synthetic lethality and induced synthetic lethality In normal cells or tumor cells without mutation within the homologous recombination repair or Wnt pathways, PARP and Wnt signaling inhibitors have minimal effect on cell proliferation. In BRCA1/2 mutant cells, PARP inhibitors lead to cell death. In Wnt‐addicted cancers, Porcupine inhibitors lead to cell cycle arrest and tumor growth inhibition. In Wnt‐addicted cancers, sublethal doses of Wnt‐βcatenin inhibitors induce a state of BRCAness sensitizing cells to PARP inhibitors.

Finally, the functional interaction between Wnt‐βcatenin signaling and the DNA damage pathways is distinct from the well‐established role of the Wnt pathway in progenitor cell self‐renewal. Consistent with this, synergy between Porcupine and PARP inhibitors was also observed in pancreatic cancer cell lines that harbor *RNF43* mutations but that are resistant to Wnt pathway inhibition, possibly the result of additional mutations evading a requirement for βcatenin signaling for the proliferation of these cells. This result suggests that harnessing this induced synthetic lethal interaction could be effective in the context of tumors resistant to Wnt pathway inhibitors or alternatively slow down the emergence of therapeutic resistance.
